# Autopolyploidization‐Induced Chromatin Remodeling Regulates Leaf Size Variation in *Brassica rapa*

**DOI:** 10.1002/advs.202513558

**Published:** 2025-12-03

**Authors:** Haoyuan Dong, Yanhong Liu, Yuanming Liu, Shuxin Xuan, Huanhuan Chen, Lai Wei, Guibao Zhang, Hongcui Pei, Zilong Dai, Yanhua Wang, Jinzhu Qiao, Shuangxia Luo, Xueping Chen, Yiguo Hong, Jianjun Zhao, Shuxing Shen, Zefu Lu, Aixia Gu

**Affiliations:** ^1^ College of Horticulture State Key Laboratory of North China Crop Improvement and Regulation Key Laboratory of Vegetable Germplasm Innovation and Utilization of Hebei Collaborative Innovation Center of Vegetable Industry in Hebei Hebei Agricultural University Baoding Hebei 071000 China; ^2^ State Key Laboratory of Crop Gene Resources and Breeding Institute of Crop Sciences Chinese Academy of Agricultural Sciences Beijing 100081 China; ^3^ School of Life Sciences University of Warwick Warwick CV4 7AL UK

**Keywords:** autopolyploidization, *Brassica rapa*, BrGRF13, BrARF11, chromatin remodeling, histone modifications

## Abstract

Whole‐genome duplication is a key evolutionary mechanism influencing gene regulation and trait development; however, how successive genome duplications reshape chromatin at the genome‐wide scale and thereby drive phenotypic innovation remains unclear. To dissect the effects of genome doubling on chromatin dynamics, gene expression, and associated trait differences, monoploid, diploid, and autotetraploid *Brassica rapa* L. ssp. *pekinensis* lines are generated with an identical genomic background and performed integrative analyses using ATAC‐seq, ChIP‐seq (H3K4me3, H3K27ac, H3K27me3), and RNA‐seq. By establishing this uniform ploidy series, nonlinear and stage‐specific chromatin and transcriptional reprogramming during autopolyploidization are revealed. Increased ploidy reprogrammed chromatin accessibility, characterized by reduced proximal and expanded distal regions, with effects particularly pronounced during the monoploid‐to‐diploid transition. Corresponding changes in H3K4me3 modifications near transcription start sites alter global gene expression. Numerous transcription factor genes are identified, of which *BrGRF13* and *BrARF11* are crucial regulators of leaf size and polarity during head development. Overall, this study elucidates the molecular basis by which ploidy variation drives chromatin remodeling and phenotypic divergence, providing new insights into how genome duplication shapes plant traits and informs polyploid crop improvement.

## Graphical abstract

This study demonstrates that monoploid, diploid, and autotetraploid *Brassica rapa* lines derived from the same genome exhibit distinct leaf morphology. Ploidy changes remodel chromatin accessibility and histone modifications to regulate gene expression, while key transcription factors *BrGRF13* and *BrARF11* control leaf size and polarity, driving phenotypic diversity and highlighting the role of autopolyploidization in trait variation and crop improvement.

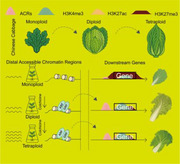

## 1 Introduction

Whole‐genome duplication (WGD) is a pivotal evolutionary force that has recurrently shaped genome evolution across kingdoms.^[^ ^1^, ^2^, ^3^ ^]^ Notably, angiosperms have universally experienced at least one WGD event, with some lineages undergoing multiple rounds of duplication.^[^ ^4^, ^5^ ^]^ For example, species in Brassicaceae experienced two WGDs,^[^ ^6^ ^]^ and *Brassicas* have also endured a unique whole‐genome triplication (WGT) event.^[^ ^7^ ^]^ Subsequently, WGD and/or WGT provide extra genomic resources, which often lead to the emergence of new genes and novel phenotypes,^[^ ^8^, ^9^ ^]^ thereby offering plants more opportunities to adapt, survive, and thrive in changing environments.^[^ ^10^, ^11^, ^12^ ^]^

Polyploids are typically divided into allopolyploids and autopolyploids. Traditionally, the effects of WGD have been largely investigated in allopolyploids. However, alterations due to heterogeneous genome dosages and interactions can complicate the interpretation of WGD consequences. Naturally occurring and artificially induced autopolyploids have attracted increasing interest in the precise evaluation of WGD.^[^ ^13^, ^14^, ^15^ ^]^ In particular, the latter possesses a more consistent genomic background.^[^ ^16^, ^17^, ^18^ ^]^ To date, most studies on plant WGD have been confined to diploid vs autotetraploid states. Additionally, the WGD impact can be accurately assessed in homozygous plants with consecutive polyploidization processes.^[^ ^19^, ^20^ ^]^

Monoploids serve as an indispensable tool in plant breeding, facilitating rapid production of homozygous lines, accelerating trait fixation, and enabling precise gene function analysis.^[^ ^21^, ^22^ ^]^ Crucially, monoploids provide a unique platform to dissect the epigenetic effects of autopolyploidization during the transition from monoploidy to diploidy. The epigenomic consequences of autopolyploidization during monoploid‐to‐diploid transitions, and their functional links to phenotypic variation—remain unknown. Moreover, how these changes evolve across consecutive autopolyploidization events is entirely unexplored.

Epigenetics provides an effective and flexible means to overcome “genomic shock” resulting from WGD.^[^ ^14^, ^23^ ^]^ In these regards, current epigenetic studies primarily focus on allopolyploid crops such as cotton (*Gossypium*),^[^ ^15^ ^]^ *Brassica napus*,^[^ ^14^, ^24^ ^]^ wheat (*Triticum aestivum*),^[^ ^25^ ^]^ and *Panax*,^[^ ^26^ ^]^ as well as several artificially induced autopolyploid species, including rice (*Oryza sativa*),^[^ ^27^ ^]^ *Arabidopsis*,^[^ ^17^, ^18^ ^]^ and *Brassica rapa*.^[^ ^28^ ^]^ For instance, dynamic DNA methylation enhances stress resistance in autotetraploid rice,^[^ ^29^ ^]^ while H3K27me3 modification plays a critical role in regulating flowering time in *Arabidopsis*.^[^ ^17^ ^]^ These studies highlight the impact of epigenetic changes on key aspects of plant trait development. However, whether and/or how these epigenetic modifications originate from autopolyploidization remains largely unknown.

*Brassica rapa* L. ssp. *pekinensis* (AA, 2n = 20) is a nutrient‐rich vegetable species closely related to *Arabidopsis* with significant economic value.^[^ ^7^, ^30^ ^]^ It has a relatively small genome (<500 Mb) and has undergone WGT, making it an ideal model for studying the effects of WGD. Following WGT, the *Brassica rapa* genome was partitioned into three subgenomes—LF, MF1, and MF2—which differ markedly in gene retention and expression.^[^ ^7^, ^31^ ^]^ Based on their homology with *Arabidopsis* genes, *Brassica rapa* genes can be classified into single‐, double‐, and triple‐homolog categories.

In this study, we generated monoploid, diploid, and autotetraploid *Brassica rapa* lines originating from the same genome. They exhibit considerable variation in key morphological traits, such as heading ability and leaf characteristics. Through multi‐omics data mining, we elucidated the impacts of two consecutive WGD events—monoploid‐to‐diploid and diploid‐to‐autotetraploid—on chromatin remodeling, genome‐wide transcription, and trait development. Unlike many previous studies that analyzed polyploid species with distinct genetic backgrounds, all lines in our study share the same genome, allowing us to directly attribute the observed epigenomic and transcriptomic changes to ploidy transitions rather than genetic variation. We revealed how WGD in monoploid, diploid, and autotetraploid lines reshapes the epigenetic landscape in a ploidy‐dependent manner. Multi‐omics analyses also identified gene sets exhibiting distinct expression patterns across ploidy levels, including key regulators of leaf development, providing new insights into how WGD drives phenotypic variation in plants. Collectively, these findings highlight the unique contribution of our study in elucidating the mechanisms through which genome doubling drives epigenetic and phenotypic diversity.

## 2 Results

### 2.1 Epigenomic Landscape of Monoploid, Diploid, and Autotetraploid *Brassica rapa*

**Figure 1:**
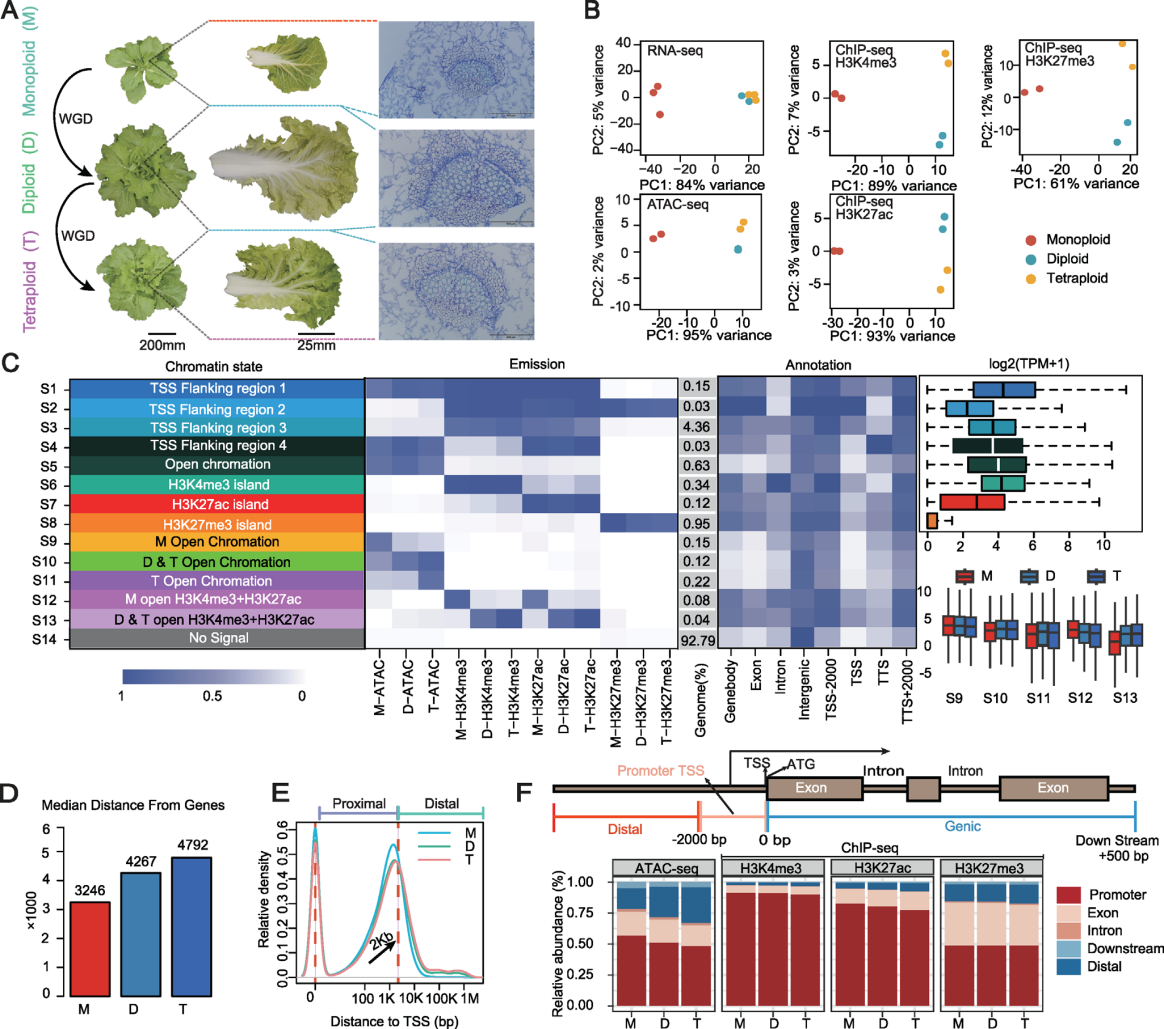
Dynamic epi‐chromatin landscapes in monoploid, diploid, and autotetraploid *Brassica rapa*. A) Leaf morphological changes during whole‐genome duplication (WGD) in *Brassica rapa*. Whole plant and single leaves were photographed at the transition stages, during which the head starts to form. Leaf sections were also photographed to show cyto‐morphological changes. Bars = 200, 25, and 0.5 mm, respectively. B) Principal Component Analysis (PCA) analysis showing reproducibility of the five omics datasets. C) The probability of chromatin accessibility and histone modifications at 14 different stages (S1–S14), along with their genomic locations and expression levels. D,E) Median distance of Accessible chromatin regions (ACRs) from genes and transcription start sites (TSS) in monoploid (M), diploid (D), and autotetraploid (T) samples. F) Distribution of ACRs and peak profiles.

The monoploid, diploid, and autotetraploid plants originated from the same genome (see Experimental Section).^[^ ^32^ ^]^ These different ploidy levels differed in leaf size, which was primarily attributed to both cell number and size (**Figures** **1**A; S1A,B, Supporting Information). During the heading formation stage, the leaves of diploid and autotetraploid plants showed distinct curvature and folding, whereas monoploid leaves remained relatively flat (Figure S1C, Supporting Information).

To further investigate the molecular mechanisms underlying these phenotypic differences, we performed a systematic analysis of multi‐omics data. Omics data among biological replicates were highly reproducible (Figures S2A and S3 and Table S1, Supporting Information). Principal Component Analysis (PCA) results showed that epigenetic histone modifications in diploid and autotetraploid samples clustered closely with each other but were distinct from those of the monoploid. Notably, 61–98% of epigenetic changes occurred during the transition from monoploid to diploid states, while changes between diploid and autotetraploid states only accounted for 2–12%, indicating a nonlinear pattern of epigenetic changes at different ploidy levels (Figure 1B).

To further characterize chromatin dynamics during autopolyploidization, we found that ≈7.21% of the genome was associated with Accessible Chromatin Regions (ACRs) and/or histone modifications. Among these regions, ≈6.61% of the whole genome was classified into S1–S8 clusters, showing conserved patterns across three ploidy levels. In contrast, ploidy‐specific chromatin features were observed in S9–S13, mainly ≈2,000 bp upstream and downstream of transcription start sites (TSS) or transcription termination sites (TTS), as well as in intergenic regions (Figure 1C). Further analysis of S10 and S13 further indicated that the autotetraploid retained most of the chromatin characteristics of a diploid; however, significant differences with the monoploid were observed.

Since chromatin accessibility is the predominant feature of regulatory elements, we further identified 26 665, 27 098, and 26 956 ACRs in monoploid, diploid, and autotetraploid samples, respectively (Tables S2–S14, Supporting Information). Notably, over half of these ACRs were located near TSS, with median distances of 3,246, 4,267, and 4,792 bp, respectively (Figure 1D,E), suggesting a potential increase in the proportion of distal ACRs (dACRs, ACRs > 2 kb to TSS and > 0.5 kb to TTS). The proportion of dACRs increased by 7.63% in the diploid compared with that in the monoploid, further increasing by 4.42% in the autotetraploid compared with that in the diploid. Meanwhile, ACRs in intergenic regions also play a regulatory role in gene expression (Figure S2B, Supporting Information). It is noteworthy that the peak proportion of H3K27ac modifications in intergenic regions increased slightly, but this change was less pronounced than chromatin accessibility in ACRs (Figure 1F). Additionally, the increase in H3K27ac shows a low correlation with H3K27me3 reduction, suggesting that the upregulation of H3K27ac may be relatively independent to some extent (Figure S4, Supporting Information). These results suggest that, during artificially induced autopolyploidization, distal regulatory elements may exhibit greater changes, reflecting the genome's dynamic regulatory properties. Specifically, dACRs showed a tendency to shift toward more distant intergenic regions, and these changes were particularly notable during the transition from monoploid to diploid states.

### 2.2 Triplicated Genes Rapidly Responded to Autopolyploidization through Epigenetic Reprogramming

**Figure 2:**
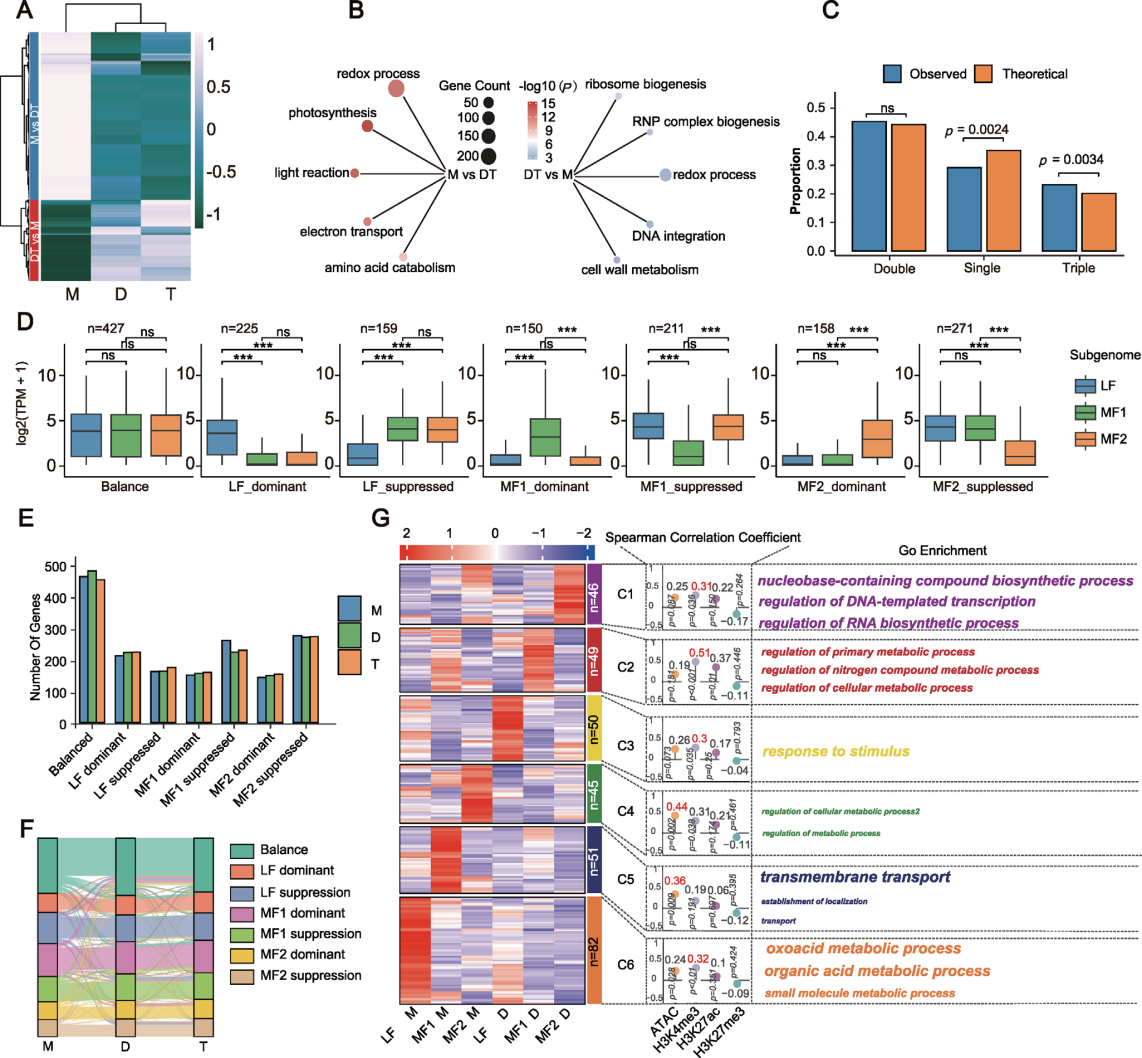
Subgenome rebalancing during genome duplication. A) Clustering differentially expressed genes (DEGs) in monoploid, diploid, and tetraploid plants. DEGs were generally clustered into M vs DT, corresponding to higher expression of monoploid over diploid and tetraploid; DT vs M, corresponding to higher expression of diploid and tetraploid over monoploid. B) GO enrichment analysis of M vs DT and DT vs M. C) Categorical data from single‐copy (*n* = 8,690; DEGs = 654), double‐copy (*n* = 11,442; DEGs = 956), and triple‐copy (*n* = 4,986; DEGs = 463) gene groups were analyzed. Pairwise comparisons yielded *p* = 0.0024 for single‐copy, *p* = 0.60 for double‐copy, and *p* = 0.0034 for triple‐copy genes (Fisher's exact test; significant at *p* ≤ 0.05; ns, *p* > 0.05). D) Gene expression levels of triads classified into seven categories—balance (*n* = 427), LF_dominant (*n* = 225), LF_suppression (*n* = 159), MF1_dominant (*n* = 150), MF1_suppression (*n* = 211), MF2_dominant (*n* = 158), and MF2_suppression (*n* = 271)—were visualized using box plots. (Mann–Whitney U test; significant at *p* < 0.05; ^***^ *p* < 0.001; ns, *p* > 0.05). E) The number of triplet genes in seven different states. F) The state transition of genes across seven states from monoploid to diploid and diploid to tetraploid. G) Clustering of DEGs between monoploid and diploid *Brassica rapa*. The correlation between expression and its epigenetic modifications is shown in the middle lane, and GO enrichment is shown in the right lane.

Autopolyploidization induces systematic transcriptome reprogramming at the whole‐genome level.^[^ ^33^ ^]^ Of the 46,479 protein‐coding genes, 4,550 were differentially expressed (|log2FC| ≥ 1, FDR ≤ 0.05, Tables S15–S20, Supporting Information), with the most (3,246 genes) showing expression changes between monoploid and diploid, whereas only 85 genes differed between diploid and autotetraploid (Figure S5, Supporting Information). Subsequently, we classified the differentially expressed genes (DEGs) into two groups: DTvM (1,485 genes with higher expression in diploid and autotetraploid) and MvDT (3,065 genes with higher expression in monoploid). The number of MvDT genes was twice that of DTvM genes (**Figure** **2**A; Tables S21–S22, Supporting Information), indicating more pronounced differences between monoploid and diploid. Gene Ontology (GO) enrichment analysis showed that DTvM genes were mainly involved in ribosome biogenesis, while MvDT genes were enriched in photosynthesis‐related processes (Figure 2B).

To verify the generality of these findings and exclude the potential influence of a single genetic background, we constructed a more comprehensive transcriptome dataset comprising three independent monoploid genotypes, three diploid genotypes, and one tetraploid *Brassica rapa*, and integrated previously published diploid and tetraploid transcriptome data.^[^ ^34^ ^]^ The integrated analyses consistently demonstrated that, regardless of genetic background, the transition from monoploid to diploid was accompanied by a much stronger transcriptional reprogramming than the transition from diploid to tetraploid, suggesting that the impact of genome doubling on transcriptomic reconfiguration is closely dependent on the ploidy stage involved (Figure S6, Supporting Information).

To investigate the relationship between subgenome bias and histone modifications following autopolyploidization, 25,118 genes were mapped to the LF, MF1, and MF2 subgenomes (Figure S7A and Table S23, Supporting Information). The LF subgenome consistently exhibited higher expression levels across all ploidy levels, accompanied by stronger chromatin accessibility and enrichment of H3K4me3 and H3K27ac marks, whereas H3K27me3 levels remained low (Figure S7B,C, Supporting Information). This trend was stable across monoploid, diploid, and autotetraploid plants, suggesting that subgenome dominance is maintained during early stages of autopolyploidization.

Notably, compared with the proportion of genes with different copy numbers in the whole genome, the number of “one‐to‐one” type DEGs decreased, the number of “one‐to‐two” type DEGs remained unchanged, and the number of “one‐to‐three” type DEGs significantly increased (Figure 2C). This indicates that the “one‐to‐three” genes are more likely to respond to autopolyploidization and subsequently undergo neo‐ and/or sub‐functionalization. After further assessing the relationship between triad expression‐biased genes and epigenetic modifications, we classified them into seven categories (Figure S7D, Supporting Information). Across monoploid, diploid, and autotetraploid states, the “balanced” category had the highest number of genes, averaging 464, accounting for 27.9% of the total triplet genes. In contrast, the number of genes in the other six categories was relatively small, ranging from 100 to 300. In the LF‐dominant subgenome, the number of LF‐dominant genes was, on average, 31.7% higher than that of LF‐suppressed genes. However, in the MF1‐ and MF2‐subgenomes, the proportion of dominant genes was, on average, ≈68.2% lower than that of suppressed genes (Figure 2D,E). These findings suggest that, after autopolyploidization, most triplet genes exhibit a certain degree of expression synchronization, implying that the expression and function of homologous triplet genes may be genetically coordinated.

Autopolyploidization caused expression state changes in 25.8% (429/1,662) and 14.8% (246/1,662) of genes during monoploid–diploid and diploid–tetraploid transitions, respectively (Figure 2F). Cluster analysis divided DEGs from the monoploid–diploid transition into six functional groups (C1–C6) (Figure 2G). Among these, genes with higher expression in the monoploid were mainly involved in oxoacid metabolism, transmembrane transport, and cellular metabolic regulation, while genes with higher expression in the diploid were enriched in nucleotide synthesis, metabolic regulation, and stress response. Further analysis showed C1–C3 and C6 groups strongly correlated with H3K4me3 modifications, while C4–C5 groups were primarily regulated by chromatin accessibility. Overall, chromatin structure and epigenetic modifications play a key role in regulating gene expression across different subgenomes, indicating that autopolyploidization not only induces changes in gene expression but also achieves selective activation or repression through chromatin remodeling with high regulatory precision.

### 2.3 WGD Coordinated Transcriptional Reprogramming through H3K4me3 and H3K27ac Modifications

**Figure 3:**
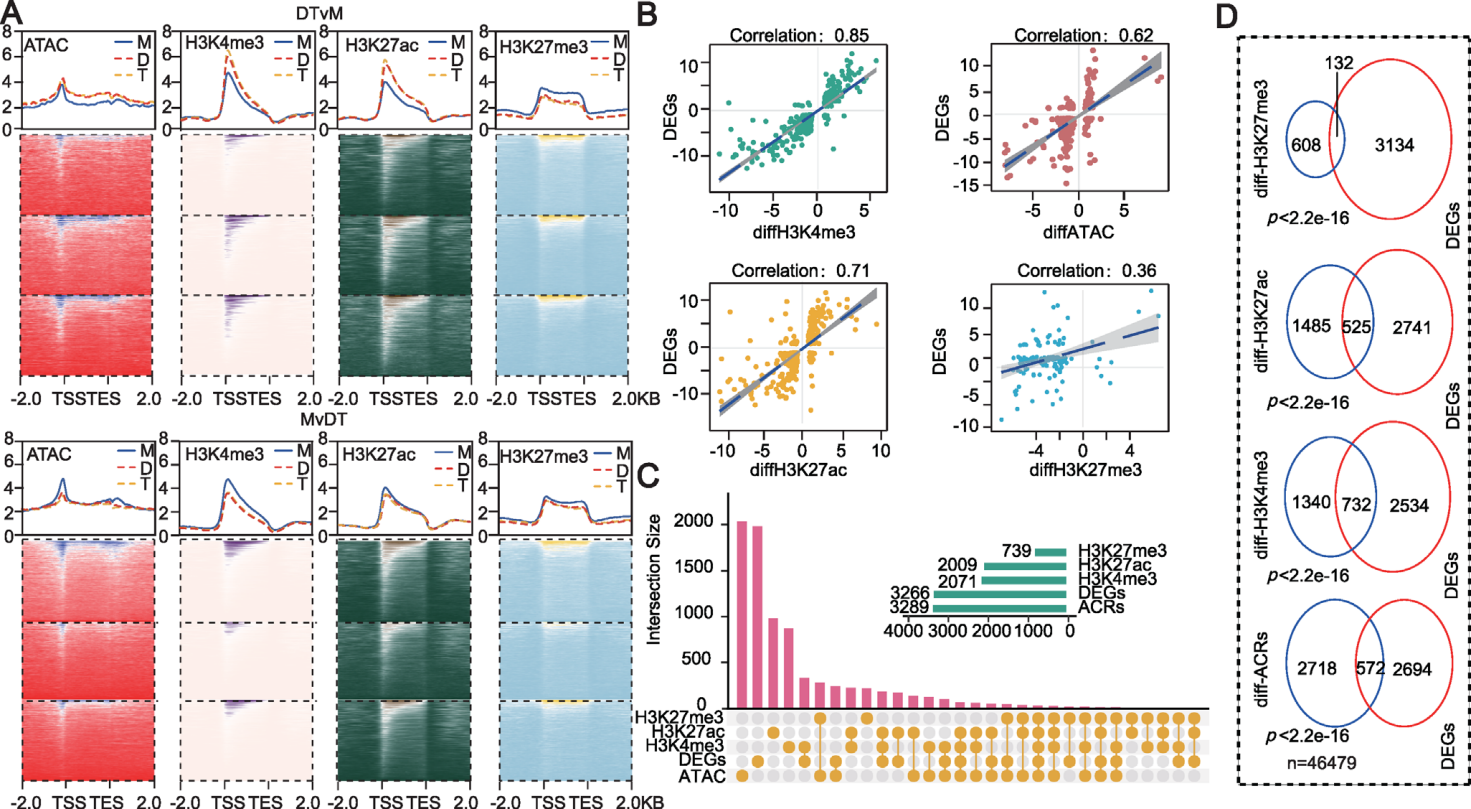
Transcriptomic variations were associated with epigenetic modification shifts during genome duplication in *Brassica rapa*. A) Chromatin accessibilities and H3K4me3, H3K27ac, and H3K27me3 modification levels of DTvM and MvDT differentially expressed genes (DEGs) in monoploid, diploid, and tetraploid plants. B) Correlation of DEG expression with chromatin accessibility and H3K4me3, H3K27ac, H3K27me3. Expression and epigenetic modification levels between monoploid and diploid plants. C) Number of overlaps between epigenetic marks and DEGs. D) A Venn diagram was generated to illustrate the overlap among DEGs and genes linked to differential histone modifications and differential ACRs. (Fisher's exact test; significant at *p* ≤ 0.05).

After WGD, chromatin may undergo epigenetic reorganization, which in turn affects gene expression.^[^ ^15^, ^35^, ^36^ ^]^ DEGs with high expression levels are frequently enriched in ACRs marked by active histone modifications, such as H3K27ac and H3K4me3, indicating that these epigenetic marks play crucial roles in maintaining high transcriptional activity (**Figure** **3**A). The repressive mark H3K27me3 is consistently observed in monoploids; however, it does not always correlate with the mRNA levels of DEGs. Notably, differences in ACR distribution and histone modification levels between diploids and autotetraploids were limited, which aligns with the relatively small number of DEGs identified between these ploidy levels. This suggests that the major transcriptomic reprogramming triggered by autopolyploidization primarily occurs during the transition from monoploid to diploid. Among various histone modifications, H3K4me3 showed the highest correlation with DEGs expression changes (Figure 3B), highlighting its central role in autopolyploidization‐induced transcriptional regulation.

Of the 3,266 DEGs identified, the vast majority were associated with changes in chromatin accessibility, H3K4me3, or H3K27ac modifications, with H3K4me3‐linked genes being the most abundant (732 genes). Importantly, a substantial number of DEGs were co‐regulated by both H3K4me3 and H3K27ac (Figure 3C,D; Table S24, Supporting Information). These findings underscore the cooperative role of H3K4me3 and H3K27ac in the transcriptional regulatory network induced by autopolyploidization and suggest that autopolyploidization fine‐tunes gene expression through the remodeling of specific histone modifications, thereby contributing to functional coordination and genomic stability.

### 2.4 Autopolyploidization Regulated Leaf Size via Growth‐Regulating Factor (GRF)–Mediated Cell Proliferation in *Brassica rapa*

**Figure 4:**
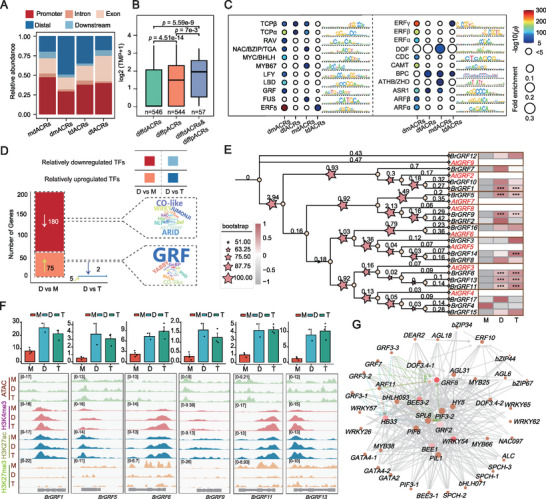
Activation of GRF TFs during genome duplication in *Brassica rapa*. A) Genomic distribution of genome duplication‐related diffACRs. mdACRs indicate ACRs that are independently open in monoploids compared with those in diploids; dmACRs indicate ACRs that are independently open in diploids compared with those in monoploids. dtACRs indicate ACRs that are independently open in diploids compared with those in tetraploids. tdACRs indicate ACRs that are independently open in tetraploids compared with those in diploids. B) Box plots were generated to illustrate expression changes of genes associated with distal differential ACRs (diffdACRs), proximal differential ACRs (diffpACRs), and genes associated with both distal and proximal differential ACRs (diffdACRs & diffpACRs). Sample sizes were *n* = 546, 544, and 57, respectively. (Mann–Whitney U test; significant at *p* ≤ 0.05). C) Bubble plots showing the enrichment of motifs across different groups of diffACRs during monoploid‐to‐diploid and diploid‐to‐tetraploid transitions. *p* < 1e‐5. D) Comparison of differentially expressed transcription factors (TFs) between diploid vs monoploid and diploid vs tetraploid, along with enrichment analysis showing the proportion of differentially expressed TFs relative to the total number within each TF family. The greater the number of differentially expressed TFs, the larger the font size used to represent the TF family. E) Phylogenetic tree showing the relationship between *Arabidopsis* and *Brassica rapa*. Growth‐Regulating Factor (GRF) families. The expression levels of these GRFs are shown on the right. Asterisks (^*^) indicate significant expression differences with a fold change ≥ 2 and FDR ≤ 0.05. F) IGV screens displaying the chromatin accessibility and histone modifications of six differentially expressed GRF genes across different *Brassica rapa* ploidy levels. G) Interaction network of TFs upregulated during the monoploid–diploid transition.

Autopolyploidization led to numerous phenotypic changes and new traits in *Brassica rapa* (Figures 1A; S1, Supporting Information). To uncover the genetic and epigenetic basis underlying *Brassica rapa* development resulting from autopolyploidization, we identified differential ACRs (diffACRs) from monoploid vs diploid and from diploid vs autotetraploid, and then integrated transcriptomic data to analyze motif enrichment within these regions and the expression changes of related transcription factors (TFs). A total of 7,413 diffACRs were obtained, of which 6,182 originated from the comparison between monoploid vs diploid (mdACRs & dmACRs) and 1,231 originated from the comparison between diploid vs autotetraploid (dtACRs & tdACRs) (**Figure** **4**A). diffACRs mapping revealed that, after autopolyploidization, intergenic regions were enriched in dmACRs and tdACRs, nearly twice as many as mdACRs and dtACRs. This indicates a progressive increase in the openness of intergenic regions in monoploid vs diploid vs autotetraploid. Additionally, ACRs with both distal and proximal differences exhibited a greater impact on gene expression compared with those with differences confined to either the proximal or distal ends (Figure 4B), highlighting the significant role of intergenic regions in the response to autopolyploidization. Further identification of motifs in diffACRs showed that ethylene response factors (ERFs), auxin response factors (ARFs), and GRFs were significantly enriched in diffACRs (Figure 4C). Meanwhile, we also observed enrichment of the DNA‐binding motif associated with the One Finger (DOF) family of TFs in autopolyploidization‐repressed ACRs. Notably, DOF TFs are involved in stress responses, plant growth, and organ (leaf, flower, and vascular tissue) development.^[^ ^37^ ^]^

We identified 229 differentially expressed TFs (Table S25, Supporting Information), among which the GRF family was significantly enriched (Figure 4D), and 16 of 17 GRF TFs were highly expressed in diploid compared with those in monoploid, with 6 significantly upregulated (Figure 4E). The expression of these GRF TFs was positively associated with the H3K4me3 and H3K27ac modifications and chromatin accessibility to a lesser extent (Figure 4F). Further study showed that these genes can be divided into two subfamilies: one comprising *BrGRF1*, *BrGRF5*, and *BrGRF9*, and the other including *BrGRF6*, *BrGRF11*, and *BrGRF13*, which are orthologs of *Arabidopsis AtGRF3* (Figure 4E). *AtGRF3* is a key regulator of leaf size, primarily acting by promoting cell proliferation.^[^ ^38^, ^39^ ^]^ In the TF regulatory network, we observed significant enrichment of interactions between GRF family TFs and *HB33* (Figure 4G), suggesting that the GRF family may participate in leaf development regulation through cooperation with *HB33*.

### 2.5 *BrGRF13* and *BrARF11* Contributed to Leaf Morphology Variation during the Heading Transition Stage in *Brassica rapa*

**Figure 5:**
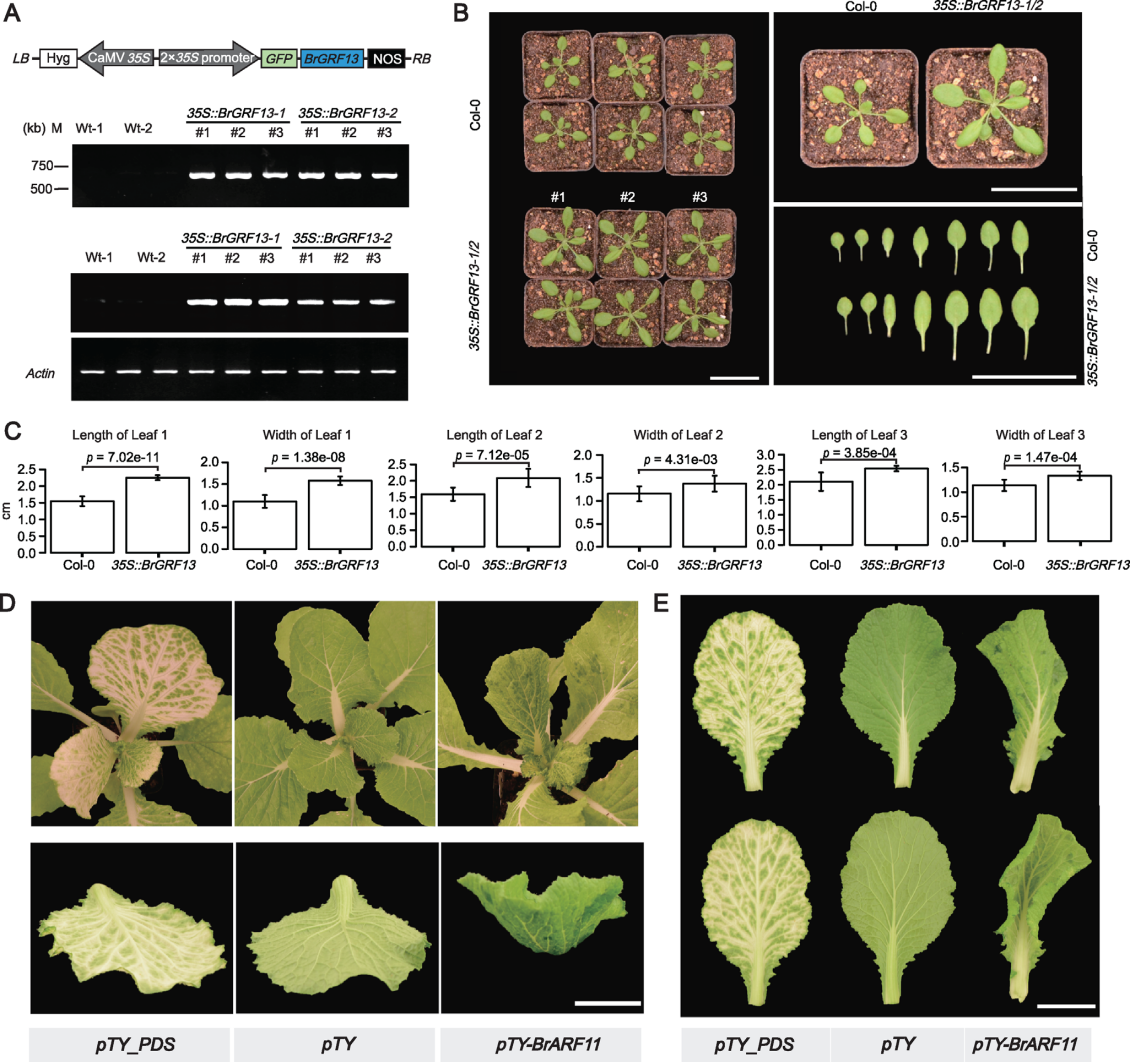
*BrGRF13* regulates leaf sizes, and *BrARF11* regulates leaf polarity. A) Construction of *2×35S‐Pro::BrGRF13* overexpressing lines in *Arabidopsis* and semi‐qRT‐PCR comparison of *BrGRF13* expression levels between transgenic and untransformed wild‐type plants, with numbers above indicating the line numbers of *BrGRF13* overexpressing plants. B) Phenotypes of wild‐type *Arabidopsis* and *BrGRF13* overexpressing plants. Scale bar = 50 mm. C) Leaf length and width were measured in Col‐0 and *2×35S‐Pro::BrGRF13* transgenic plants (*n* = 12 plants per genotype). Data are presented as mean ± SD (two‐tailed unpaired Student's *t*‐test; significant at *p* ≤ 0.05). D) *BrARF11* VIGS:*pTY‐BrARF11* represents *cis*‐silenced plants, *pTY* represents plants transformed with an empty vector, and *pTY‐PDS* represents plants transformed with the PDS whitening gene. The leaves below represent the curvature of phenotypic leaves. Scale bar = 30 mm. E) Leaf phenotypes of *BrARF11*‐VIGS plants showing curling on the adaxial side. Scale bar = 30 mm.

To identify the key TFs involved in regulating leaf morphogenesis during autopolyploidization more precisely, we focused on TF binding sites enriched during the transition from non‐heading monoploid to head‐forming diploid, which was accompanied by an upregulation of corresponding TF expression levels. A total of 68 TFs were identified, among which *BrARF11* (a member of the ARF family) and six GRF family members (*BrGRF1, BrGRF5, BrGRF6, BrGRF9, BrGRF11*, and *BrGRF13*) were significantly enriched. These genes were expressed at significantly higher levels in diploid vs monoploid. Subsequently, we selected *BrGRF13*, a homolog of *AtGRF3*,^[^ ^38^, ^39^ ^]^ for further investigation. *BrGRF13* showed its highest expression in diploid plants compared with monoploids, and was located within the dominant subgenome LF of triplet genes. We transformed *Arabidopsis* Col‐0 with a ‘CaMV 35S promoter‐driven *BrGRF13* expression cassette and generated two overexpression lines (**Figure** **5**A,B), OE‐1, OE‐2. Semi‐qRT‐PCR showed that the expression levels of *BrGRF13* in the OE‐1 and OE‐2 lines were significantly higher than those in non‐transformed Col‐0 under normal growth conditions. Subsequently, we measured the leaf sizes and found that the leaf length and width of the OE‐1 and OE‐2 lines increased by approximately 30%, or 50%, respectively (Figure 5C). These findings indicated that *BrGRF13* positively regulates plant leaf size, consistent with the observation that diploid leaves were larger than monoploid leaves in *Brassica rapa* (Figures 1A; S1A, Supporting Information).

Similarly, *BrARF11*, an ortholog of *AtARF11* that regulates leaf polarity,^[^ ^40^ ^]^ was highly expressed in diploid *Brassica rapa*. We identified S169 from the mutant population of *Brassica rapa*.^[^ ^41^ ^]^ Unlike wild‐type *Brassica rapa* A03, mutant S169, containing a G to A point mutation that affects alternative splicing of *BrARF11* pre‐mRNA, exhibited a non‐heading phenotype (Figure S8, Supporting Information). In addition, after silencing *BrARF11* through virus‐induced gene silencing (VIGS), we found that *BrARF11*‐VIGSed plants exhibited curled dorsal leaves (Figures 5D,E; S9A,B, Supporting Information), opposite to the inward curling of diploid leaves during head formation. This indicates that high *BrARF11* expression could lead to leaf curling toward the adaxial side, whereas low expression results in curling toward the abaxial side, suggesting that *BrARF11* is involved in the development of leaf polarity during autopolyploidization.

## 3 Discussion

How WGD affects gene expression and the formation of phenotypes (i.e., new traits) during evolution is one of the long‐standing questions in biology. The impact of autopolyploidization on genome‐wide transcriptional regulation is often perceived to be limited in diploid vs autotetraploid states. For example, only a small number of DEGs were identified in yeast undergoing WGD.^[^ ^42^ ^]^ Similar phenomena have also been observed in rice,^[^ ^16^ ^]^ potato (*Solanum tuberosum*),^[^ ^19^ ^]^ maize (*Zea mays*),^[^ ^20^ ^]^ and *Brassica rapa* (this study). However, unexpectedly, autopolyploidization from monoploid to diploid states resulted in significant chromatin remodeling and transcriptional regulation, leading to the establishment of new phenotypes in *Brassica rapa*. Further analysis of DEGs across three monoploid, three diploid, and one tetraploid genotypes revealed that transcriptional remodeling primarily occurs during the transition from monoploid to diploid. Although the number of DEGs between diploid and tetraploid slightly increased compared with autopolyploids, it remained far lower than that observed between monoploid and diploid (Figure S6, Supporting Information). This suggests that the impact of polyploidization on gene expression is not a simple linear accumulation but is particularly pronounced at specific ploidy stages. In contrast, genome doubling from diploid to tetraploid causes relatively minor perturbations in the overall transcriptome, possibly reflecting the establishment of stability and buffering mechanisms in the diploid genome following the first round of genome doubling. These observations also imply that polyploidization primarily influences the formation of new traits during a “sensitive window,” rather than merely through a straightforward increase in gene dosage. The effects of polyploidization on genetic and epigenetic control of genome‐wide gene expression, as well as the relevant consequences of phenotypic changes, may have been underestimated. Furthermore, our findings show that autopolyploidization functions in a ploidy‐dependent manner to influence gene transcription and trait development. By studying autopolyploidization in *Brassica rapa* with the same genotype at three different ploidy levels, this work deepens our understanding of autopolyploidization and provides (epi)genetic and molecular bases to use monoploids in crop breeding.

Chromatin accessibility reprogramming has been extensively investigated in various plants, including crops, yet chromatin reprogramming patterns in response to autopolyploidization remain poorly understood. In the present study, we analyzed the chromatin dynamics of monoploid, diploid, and autotetraploid *Brassica rapa* derived from the same genomic background. We found that genomic dosages can drive the emergence of dACRs and alter the ratio of dACRs vs pACRs to regulate the expression of nearby genes. Thus, even without changes in genome size, autopolyploidization can influence the proportion of distal regulatory elements to modulate gene expression. This differs from the perception that the appearance of dACRs might be linked to genome size.^[^ ^43^ ^]^ Our findings are also supported by the fact that the proportion of dACRs increases after allopolyploidization in cotton.^[^ ^44^ ^]^ In addition, with autopolyploidization, both chromatin accessibility and H3K27ac levels increased. Although the number of distal H3K27ac regions also increased, the magnitude of change did not exceed that of chromatin accessibility. While some regions with increased H3K27ac were associated with the loss of H3K27me3, most H3K27ac changes occurred independently, indicating that alterations in H3K27ac and H3K27me3 are largely independent during autopolyploidization.

One of the notable genomic characteristics of *Brassica* species is the existence of various subgenomes along with nuclear chromosomes and subgenome dominance. In *Brassica rapa*, expression of genes in the less vs more fractioned subgenomes (LF vs MF1 and MF2) is significantly high, establishing LF as the primary subgenome, i.e., LF dominance.^[^ ^7^ ^]^ Such LF dominance is closely related to biased distributions of transposons and their cognate 24‐nt small RNAs, with the latter being associated with RNA‐directed DNA methylation.^[^ ^31^ ^]^ However, whether and how autopolyploidization might shape subgenome dominance in autopolyploids remains largely obscure. The current study revealed that the LF subgenome in monoploid, diploid, and autotetraploid *Brassica rapa* is characterized by marked increases in H3K4me3 and H3K27ac, along with evident decreases in H3K27me3 and strong chromatin accessibility, suggesting that LF dominance is influenced by epigenetic modifications. Furthermore, once subgenome dominance is established in autopolyploidy, short‐term autopolyploidization events do not significantly alter its dominant position. This suggests that the establishment of LF dominance is likely a gradual process, differing from the rapid subgenome differentiation due to transposon activity and genetic incompatibility between subgenomes in allopolyploids. After autopolyploidization, the proportion of DEGs was significantly higher in three‐copy genes than in single‐copy genes. These dynamic changes in the expression of multicopy genes may disrupt the original expression equilibrium, leading to the formation of novel transcriptomic profiles or gene functions.^[^ ^45^ ^]^ This suggests that three‐copy genes are particularly susceptible to genomic dosage effects, and their expression changes may reflect the process of genomic adjustment and adaptation, thereby driving the evolution of new species following autopolyploidization.

The underlying mechanisms driving the regional bias of dACRs following autopolyploidization remain unclear. However, our study revealed that diffACRs during autopolyploidization are primarily mapped to transcriptional regulatory elements associated with ERF, ARF, BPC, and GRF family genes. Indeed, several members of the GRF family genes displayed altered expression. Notably, in poplar (*Populus*), *PpnGRF6* reportedly modulates leaf sizes in diploids and triploids.^[^ ^46^ ^]^ This suggests that GRF family genes may be affected by polyploid events in different plant species. In *Brassica rapa*, autopolyploidization can exert simultaneous impacts on the GRF family members *BrGRF6*, *BrGRF11*, and *BrGRF13*, all homologous to *AtGRF3*. This may be related to the spatial proximity of these homologous genes throughout Brassicaceae evolution.^[^ ^47^ ^]^ Nevertheless, as evidenced by the involvement of GRF family genes in leaf morphogenesis and heading formation, our results demonstrate that autopolyploidization‐directed chromatin remodeling can affect gene expression to develop new traits in *Brassica rapa*.

In conclusion, by leveraging monoploid, diploid, and autotetraploid *Brassica rapa* as a unique experimental system coupled with multi‐omics analyses, this study advances our understanding of how autopolyploidization alters ploidy‐dependent dynamics of chromosome‐wide histone modifications, global chromatin accessibility, and transcriptomic profiles. These changes collectively contribute to the epigenetic modulation of gene expression, subgenome dominance, genomic evolution, and new trait development in *Brassica rapa*, with potential relevance to other plant species.

## 4 Experimental Section

### Plant Materials and Growth Conditions

The monoploid was generated via isolated microspore culture from the inbred line ‘85‐1’.^[^ ^32^ ^]^ Subsequently, the diploid plant was obtained by treating the monoploid with colchicine to induce chromosome doubling, and the autotetraploid plant was produced by further colchicine treatment of the diploid plant. To maintain consistent growth conditions across all ploidy levels, all plant materials were synchronously propagated through plant tissue culture in the preceding generation and uniformly treated with rooting medium before transplanting. On August 10th, 2021, all experimental materials were transplanted simultaneously into plastic greenhouses at the experimental station of Hebei Agricultural University (Baoding, Hebei Province, China; 115.47°E, 38.87°N). At the heading transition stage (60 days after sowing),^[^ ^48^ ^]^ the second or third young leaf from the inside out was collected between 10:00 and 11:00 am. For the epigenetic analyses, two biological replicates were adopted for each ploidy level, each consisting of five plants from the corresponding ploidy group. All collected samples were immediately flash‐frozen in liquid nitrogen and stored at −80 °C for subsequent data analysis. Three independent monoploid genotypes, three diploid genotypes, and one tetraploid genotype were grown on MS medium. Leaves of uniform size were sampled, with three plants of the same genotype pooled per sample, and immediately stored at −80 °C for RNA extraction. Two biological replicates were prepared for the experiment.

### Library Preparation and Sequencing for RNA‐seq, ATAC‐seq, and ChIP‐seq

Total RNA was extracted using TRIzolTM Reagent (Invitrogen, 15596‐026). The RNA‐Seq library was constructed by Berry Genomics (Beijing, China), and sequenced with the Illumina NovaSeq 6000 platform. All sequencing data were 150bp paired‐end reads. The RNA‐seq libraries for the three independent monoploid genotypes, three independent diploid genotypes, and one tetraploid genotype were constructed using the VAHTS Universal V10 RNA‐seq Library Prep Kit for Illumina (Vazyme, China) following the manufacturer's instructions, with index codes added to each sample. The libraries were purified using VAHTS DNA Clean Beads (Vazyme, China), and library concentrations were measured with a Qubit 3.0 Fluorometer (Invitrogen, USA). Library fragment size distributions were analyzed using an Agilent 2100 Bioanalyzer (>2 nM). The dual‐indexed libraries were sequenced on the SURFSeq 5000 platform (GeneMind Biosciences Company Limited, China). ATAC‐seq was performed using our previously established protocol.^[^ ^49^ ^]^ A total of 1 g frozen sample was minced in 1 mL ice lysis buffer (15 mm Tris‐HCl pH 7.5, 20 mm NaCl, 80 mm KCl, 0.5 mm spermine, 5 mm 2‐Mercaptoethanol, 0.2% Triton X‐100). The slurry containing the nuclei extract was filtered twice through a 40 µm filter. The crude nuclei containing DAPI (Sigma, D9542) were loaded onto a flow cytometer (BD FACSCanto) for selection. The nuclei pellet was obtained by centrifugation and washed with Tris‐Mg buffer (10 mm Tris‐HCl, pH 8.0, 5 mm MgCl_2_) and Tn5 transposomes in 40 µL TTBL buffer (Vazyme, TD501) were added for a 30‐min incubation at 37 °C. Subsequently, the integration products were purified using the NEB Monarch DNA Cleanup Kit (T1030S), and library amplification was performed using NEB Next Ultra II Q5 master mix (M0544L). The amplified libraries were purified using Hieff NGS DNA Selection Beads (Yeasen, 12601ES03). ChIP‐seq was performed as previously described using antibodies against H3K27me3 (Abcam, ab6002),^[^ ^50^ ^]^ H3K4me3 (Millipore, 07–473), and H3K27ac (Abclonal, A7253). Libraries were constructed using the Kit (TransGen Biotech, KP201‐02) and sequenced by Annoroad Gene Technology (Beijing, China). All libraries were sequenced on the Illumina NovaSeq 6000 platform to produce 150‐bp paired‐end reads.

### Data Preprocessing

Raw reads from RNA‐seq, ATAC‐seq, and ChIP‐seq were trimmed using fastp (v0.21.0).^[^ ^51^ ^]^ RNA‐seq reads were aligned to the reference genome using HISAT2 (v 2.1.0).^[^ ^52^ ^]^ ATAC‐/ChIP‐seq reads were aligned with Bowtie2 (v2.11.1),^[^ ^53^ ^]^ and duplicates were removed using Picard (v2.16.0, https://broadinstitute.github.io/picard). Only reads with MAPQ >10 were kept (SAMtools v1.3.1).^[^ ^54^ ^]^

### ATAC‐seq and ChIP‐seq Peak Identification

Peaks were called using MACS2 (v2.2.7.1),^[^ ^55^ ^]^ and overlapping peaks between biological replicates were considered reproducible.

### Differential Peaks and diffACRs

Merged peaks were divided into 200 bp windows with a sliding step of 50 bp. Tn5 insertion counts or ChIP coverage were normalized, and differential peaks were identified using DESeq2 with thresholds of fold change >1.5 and peak length ≥200 bp.^[^ ^56^ ^]^

### RNA‐seq Differential Expression Analysis

Gene expression was quantified using Stringtie (TPM), counted with featureCounts,^[^ ^57^, ^58^ ^]^ and DEGs were identified using DESeq2 with fold change >2 and FDR < 0.01.

### Downstream Analysis

Motif enrichment analysis of diffACRs was performed using MEME with the Jaspar database as reference,^[^ ^59^, ^60^ ^]^ using random sequences as background. Peaks or ACRs were assigned to target genes using ChIPseeker based on proximity to the nearest TSS.^[^ ^61^ ^]^ TSS‐centered visualization was generated using computeMatrix from deeptools (v3.5.1‐1).^[^ ^62^ ^]^

### Chromatin State Annotation

Chromatin states were inferred using ChromHMM based on combinatorial patterns of histone modifications (H3K27me3, H3K4me3, and H3K27ac) together with ATAC‐seq data.^[^ ^63^ ^]^ A 14‐statutes model was constructed, selected for its stability and interpretability in downstream analyses.

### Gene Expression Clustering

DEGs were grouped using *k*‐means clustering to identify major expression patterns across samples.

### Gene Copy Classification

Based on homology with *Arabidopsis thaliana* genes, *Brassica rapa* genes were classified into three copy types: 1‐to‐1, 1‐to‐2, and 1‐to‐3, corresponding to one, two, or three copies in *Brassica rapa* for each single‐copy gene in *Arabidopsis*.

### Transformation of Arabidopsis

To create transgenic plants overexpressing *BrGRF13*, the full‐length cDNA was amplified by PCR using the primers *BrGRF13*‐forward (5′‐ATGAACTATACAAAGGCGCGCCAATGGATTTGCAACTGAAGCAT‐3′) and *BrGRF13*‐reverse (5′‐GATCGGGGAAATTCGAGCTCTCAATGAAAGGCTGTGTGGA‐3′). The amplified cDNA fragment was cloned into the pMDC43 vector containing the CaMV 35S promoter to construct the plasmid CaMV35S (*35S:BrGRF13*). The pCAMBIA2300‐35S‐OCS vector without the *BrGRF13* cDNA insert was used as a negative vector control. The 35S vector and control constructs were separately transformed into Agrobacterium GV3101, and then Agrobacterium‐mediated transformation of *Arabidopsis* was performed.^[^ ^64^ ^]^ The transgenic plants were screened on 1/2 MS agar plates containing 18 mg mL^−1^ hygromycin, and homozygous T3 generation transgenic plants were used for subsequent analyses.

### Virus‐Induced Gene Silencing in *Brassica rapa*

Plants were grown in a climate chamber at a temperature of 22°C, 16/8h (light/dark), and a relative humidity of 50%. Two weeks later, *pTY* vector containing the target gene was delivered into seedlings by particle bombardment, and *pTY* and *pTY‐PDS* were used as controls.^[^ ^65^ ^]^ A total of 1 µg of DNA plasmid was coated onto gold particles and mixed with 50µL of 2.5 m CaCl_2_ and 20 µL 0.1 m spermidine sequentially. The DNA plasmid was stored on ice for 20 min and shaken with an oscillator for 30 min. Then, the particles were washed with 70% ethanol once and absolute ethanol twice, leaving 10 µL of particle suspension. Particle bombardment was implemented using a PDS 1000/He biolistic gun (BioRad, Hercules, USA). After bombardment, the plants were promptly transferred to the nutrient substrate, kept in the dark for 24 h, and then grown in a greenhouse with a temperature of 22°C, and a cycle of 16/8h (light/dark). The phenotype of seedlings was observed, and the silencing efficiency of target genes was detected by qRT‐PCR.

### Statistical Analysis

Unless otherwise specified, all statistical analyses and figure generation were performed using R software (version 4.2.2, CRAN). All experimental data were expressed as mean ± standard deviation (mean ± SD). Specific statistical methods were applied as follows: Fisher's exact test was used for categorical data analyses in Figures 2C and 3D; the Mann‐Whitney U test was applied for non‐parametric two‐group comparisons in Figures 2D and 4B; S6B and S7B (Supporting Information); Student's *t*‐test was used for mean comparisons in Figure 5C. One‐way ANOVA followed by Tukey's HSD test was used in Figure S1B. Statistical significance was defined as *p* ≤ 0.05 unless otherwise stated.

## Conflict of Interest

The authors declare no conflict of interest.

## Author Contributions

H.D. and Y.‐H.L. contributed equally to this work. A.G. and Z.L. designed the experiments. H.D., Y.‐H.L., Y.‐M.L., Z.D., and H.P. analyzed data. H.C. and G.Z. performed the molecular experiments. S.X., S.L., and X.C. involved in the planting and management of materials. L.W., J.Q., and Y.W. carried out the materials collection. H.D., A.G., and Z.L. analyzed and interpreted data as well as wrote the manuscript. Y.H., J.Z., and S.S. interpreted data and helped redraft the manuscript.

## Supporting information



Supporting Information

Supporting Information

## Data Availability

The data that support the findings of this study are openly available in NCBI Gene Expression Omnibus (GEO) at https://www.ncbi.nlm.nih.gov/geo/query/acc.cgi?acc=GSE308545, reference number 308545. RNA‐seq data from different genotypes have been deposited in the Genome Sequence Archive (GSA) under accession number CRA032230 (https://ngdc.cncb.ac.cn/gsa/).
