# Supplementary material for: Autopolyploidization‐Induced Chromatin Remodeling Regulates Leaf Size Variation in Brassica rapa
Source: Adv Sci (Weinh). 2025 Dec 3;13(9):e13558. doi: 10.1002/advs.202513558 (PMC12904041; doi:10.1002/advs.202513558)
Supplement: Supplementary file 1 — Supporting Information [file ADVS-13-e13558-s001.docx]

Supporting Information

**Autopolyploidization-Induced Chromatin Remodeling Regulates Leaf Size Variation in *Brassica rapa***

Haoyuan Dong, Yanhong Liu, Yuanming Liu, Shuxin Xuan, Huanhuan Chen, Lai Wei, Guibao Zhang, Hongcui Pei, Zilong Dai, Yanhua Wang, Jinzhu Qiao, Shuangxia Luo, Xueping Chen, Yiguo Hong, Jianjun Zhao^*^, Shuxing Shen^*^, Zefu Lu^*^, Aixia Gu^*^

**
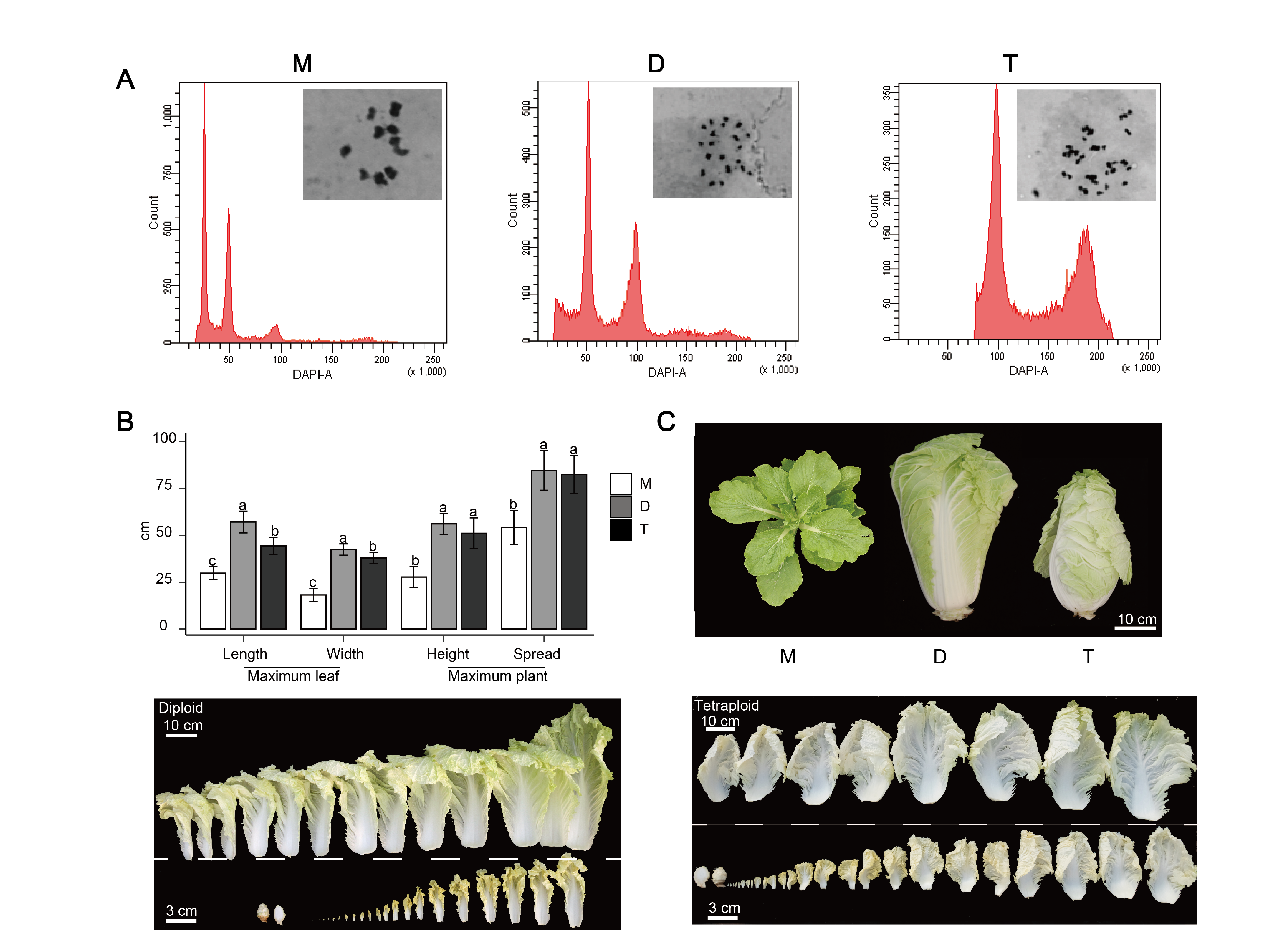
**

**Figure S1.** Trait indices of homologous monoploid, diploid, and autotetraploid *Brassica rapa*. A) The figure shows flow cytometry results for the M, D, and T from left to right. Insets show representative chromosome spreads confirming ploidy levels. B) Bar graph shows the maximum leaf length, maximum leaf width, plant height, and plant spread of M (*n* = 16), D (*n* = 13), and T (*n* = 13) *Brassica rapa*. Data are mean ± SD. Different lowercase letters indicate significant differences among groups (one-way ANOVA with Tukey’s HSD, *P* < 0.05). C) Head phenotypes of M, D, and T plants. Individual leaves from diploid and autotetraploid of *Brassica rapa* are shown.


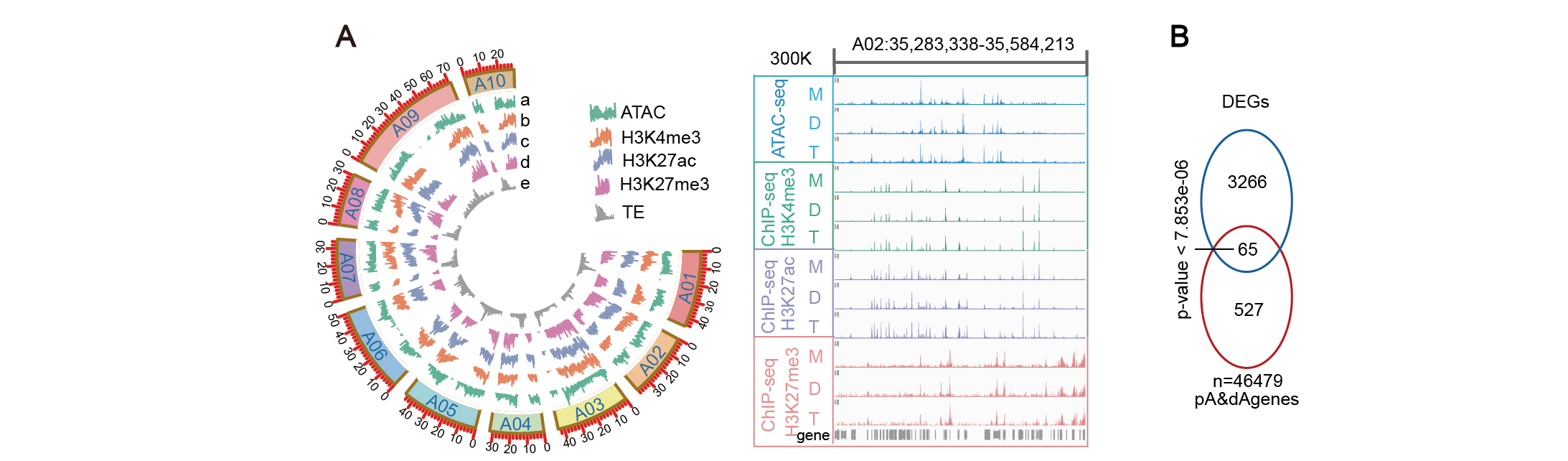


**Figure S2.** Whole-genome presentation of *Brassica rapa* with different ploidy levels. A) The circos plot displays the genome-wide chromatin accessibility and histone modification landscape of diploid *Brassica rapa*. Integrative Genomic Viewer (IGV) screens display chromatin accessibilities and histone modifications in a section of chromosome 2 of different ploidies. B) Venn diagram showing the overlap between genes associated with distal and proximal ACRs (pA&dA genes) and differentially expressed genes (DEGs) (Fisher’s exact test; significant at *p* ≤ 0.05).


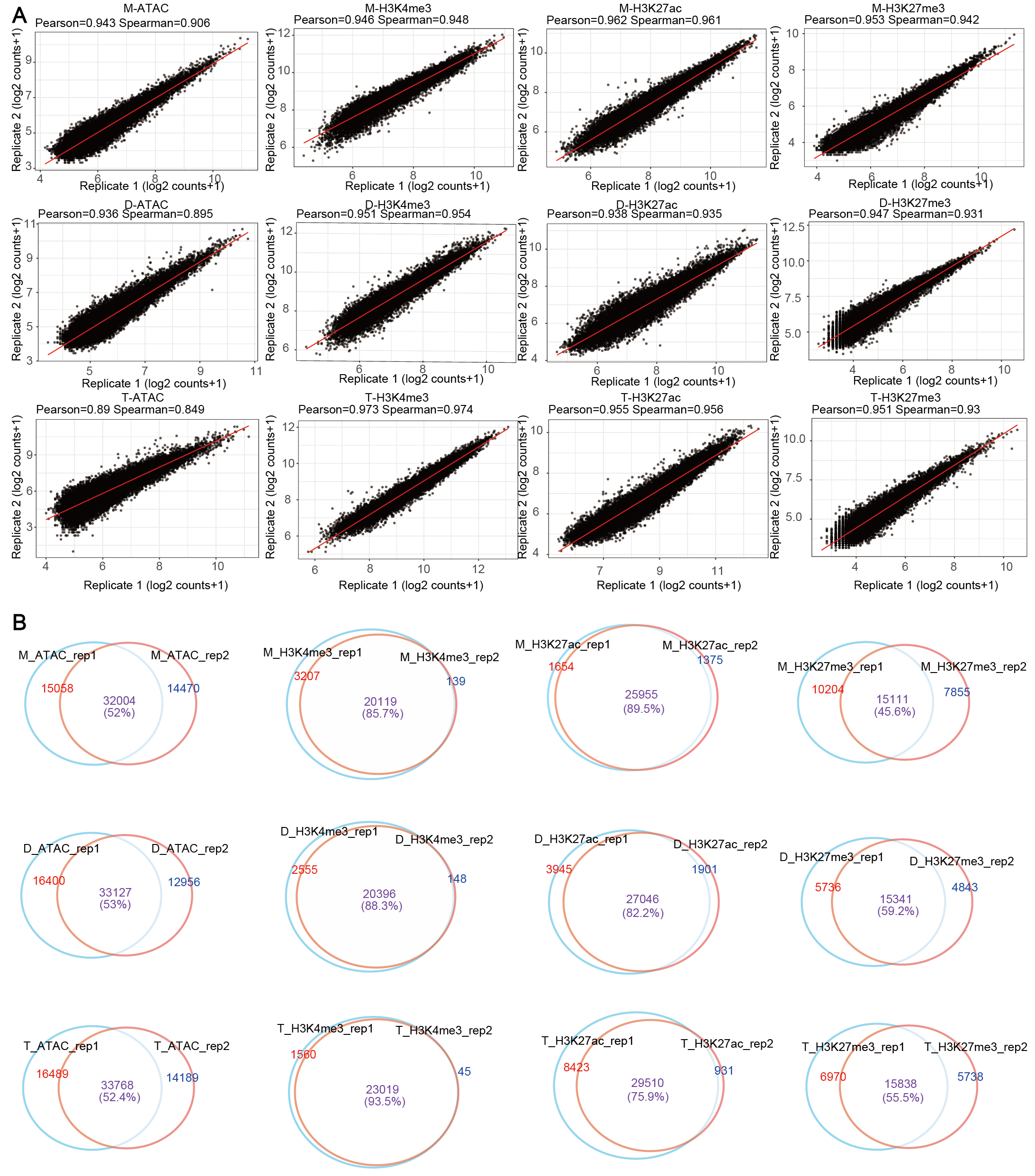


**Figure S3.** Quality control of ATAC-seq, ChIP-seq datasets. A) Correlation coefficients between two biological replicates. B) Venn diagram showing the overlap of peaks between two replicates.


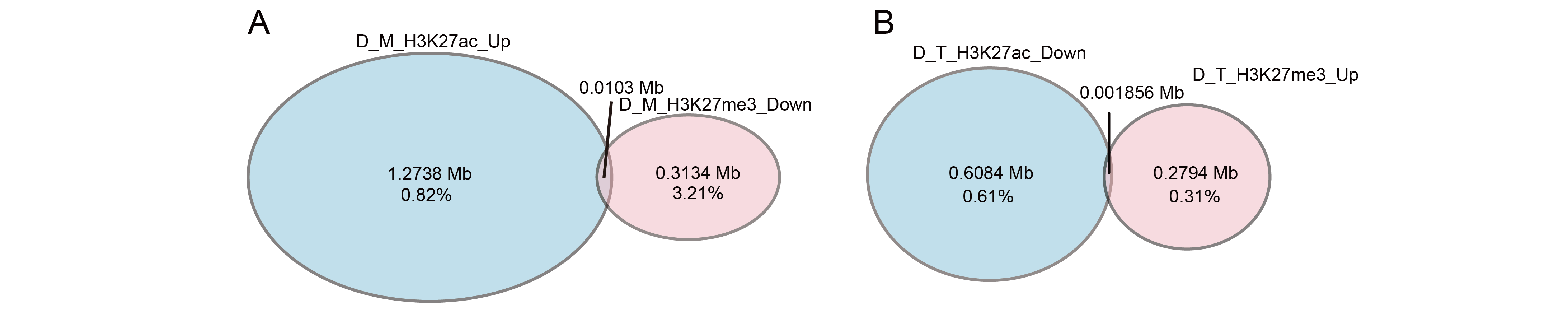


**Figure S4.** Overlap between upregulated H3K27ac and downregulated H3K27me3. A) Diploids compared to monoploids. B) Tetraploids compared to diploids.


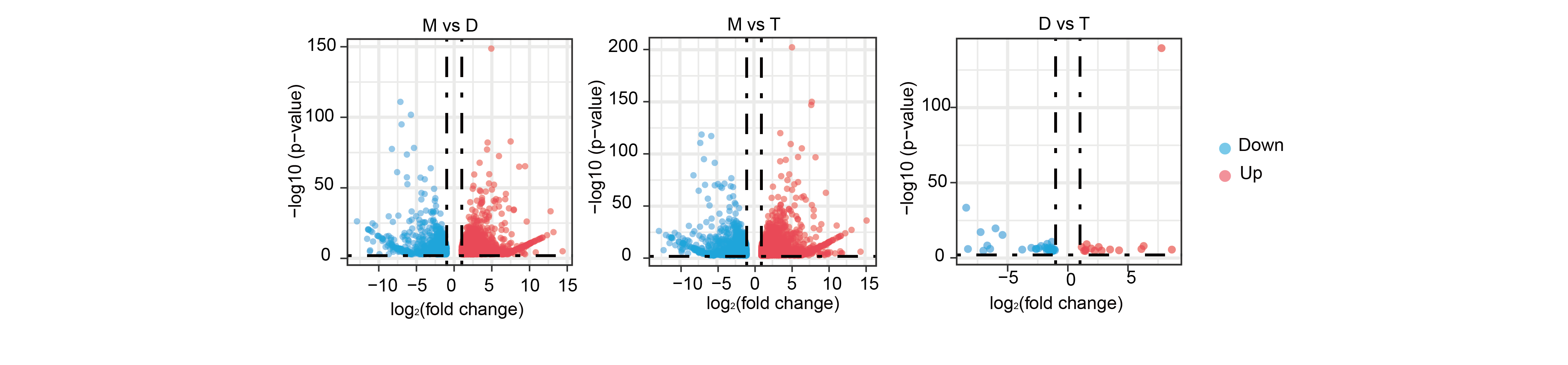


**Figure S5.** Differential transcriptome analysis of *Brassica rapa* with three ploidy levels. The volcano plot shows the number of upregulated and downregulated genes between each pair of the three M, D, and T.


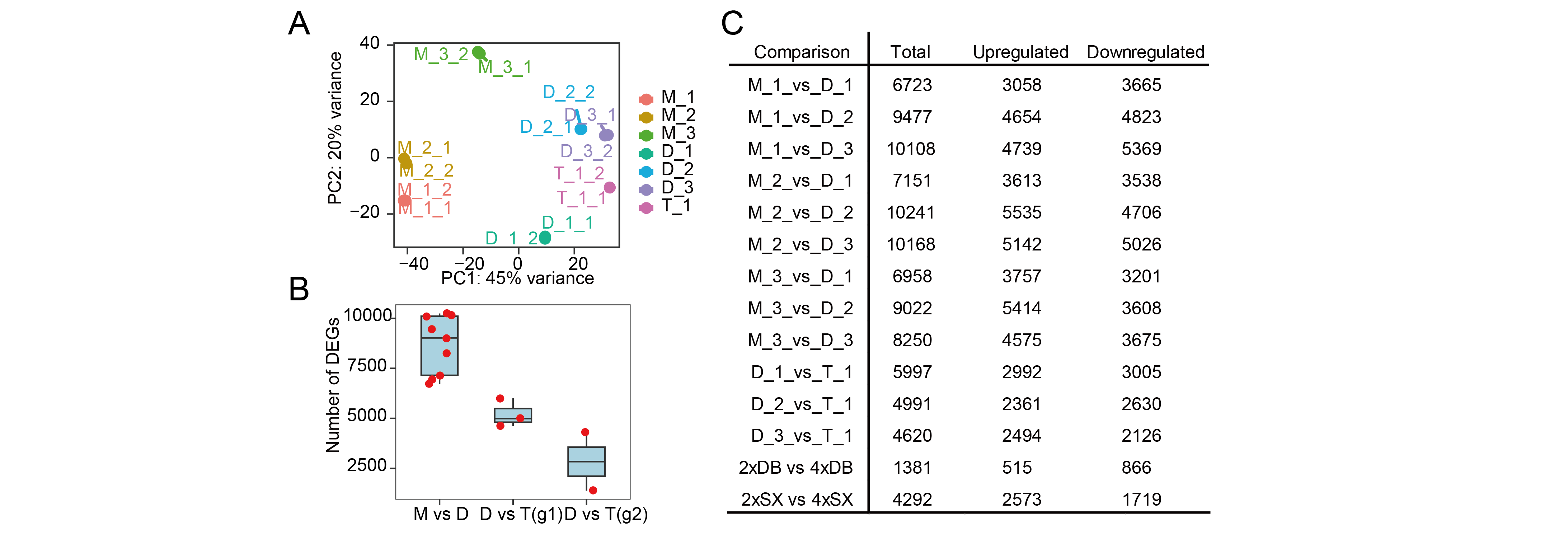


**Figure S6.** DEGs across different genotypes and ploidy levels. A) Principal component analysis (PCA) of RNA-seq samples. B) Three genotypes of monoploid and diploid samples, along with a single tetraploid sample. g1: diploid–tetraploid combination at the seedling stage; g2: diploid–tetraploid combination at the heart stage (56 days after planting). C) Two diploid samples and two tetraploid samples.


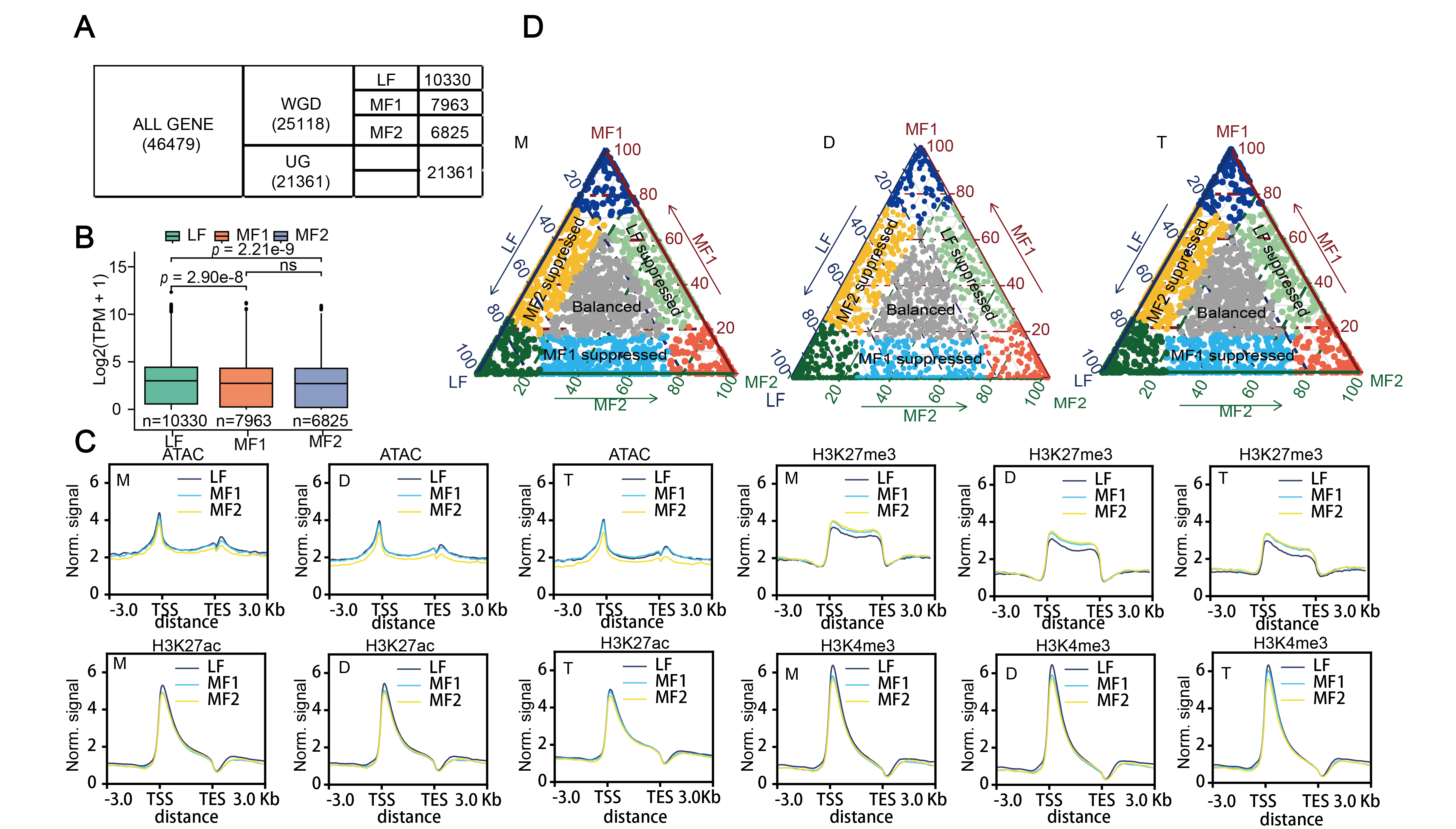
**Figure S7.** Subgenomes and epigenetic modifications of *Brassica rapa* with three ploidy levels. A) The number of subgenome genes in the genome and the counts of LF, MF1, MF2, and UG. B) Box plots were generated to show expression differences among LF, MF1, and MF2 genes (*n* = 10,330; 7,963; and 6,825, respectively) (Mann–Whitney U test; significant at *p* ≤ 0.05; ns, *p* > 0.05). C) The abundance of epigenetic signals ATAC, H3K4me3, H3K27ac, and H3K27me3 in the LF, MF1, and MF2 genes. D) A ternary plot showing the subgenome expression bias of triplet genes in the M and T ploidy materials, with the following categories: Balanced Type: Balanced expression of the triplet genes. LF Suppression: Lower expression from the LF subgenome compared to the other two triplet genes. MF1 Suppression: Lower expression from the MF1 subgenome compared to the other two triplet genes. MF2 Suppression: Lower expression from the MF2 subgenome compared to the other two triplet genes.

**
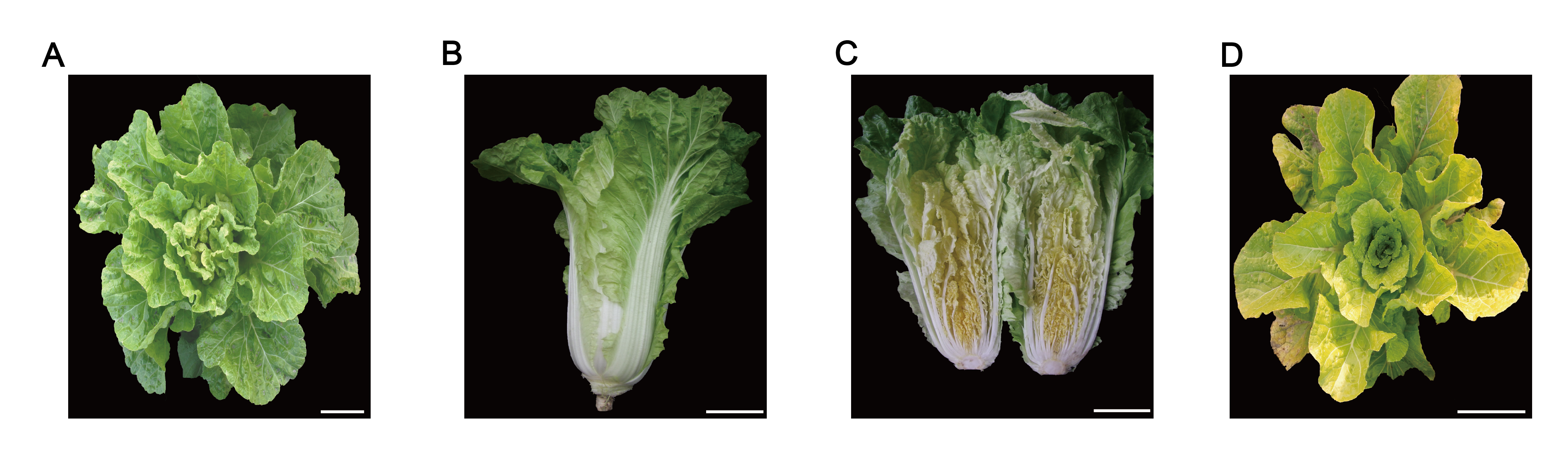
**

**Figure S8.** The phenotype of the wild type (A03) and the *brarf11* mutant (S169) at the end of heading stage. A) The heading A03. Scale bar = 10 cm. B) The leafy head of A03. Scale bar = 10 cm. C) Longitudinal cutting of A03 leafy head. Scale bar = 10 cm. D) The non-heading S169. Scale bar = 10 cm.


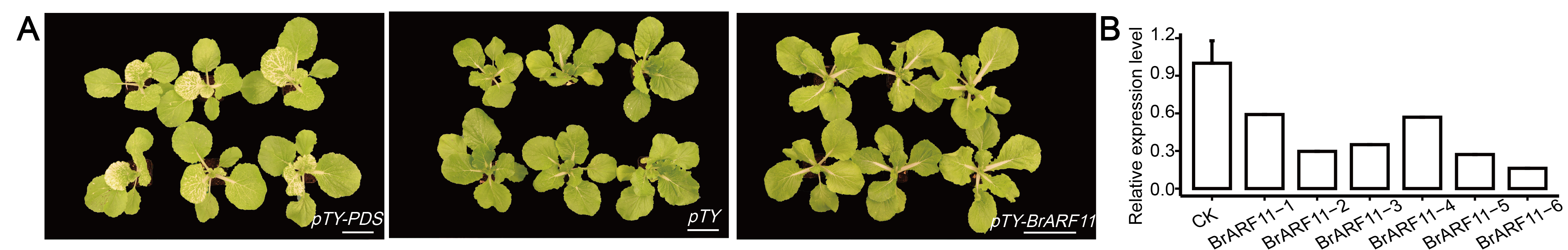


**Figure S9.** Silencing of *BrARF11* via VIGS Alters Plant Phenotype and Gene Expression. A) The phenotype after *BrARF11* VIGS shows *BrARF11* gene on the left, empty vector in the middle, and PDS gene on the right. Bar indicates 50 mm. B) The bar chart represents the relative expression levels between *BrARF11*-silenced plants and the control.
